# Clinical and Demographic Features of Vertigo: Findings from the REVERT Registry

**DOI:** 10.3389/fneur.2013.00048

**Published:** 2013-05-10

**Authors:** Sam Agus, Heike Benecke, Cornelia Thum, Michael Strupp

**Affiliations:** ^1^Abbott Products Operations AGAllschwil, Switzerland; ^2^Abbott Laboratories GmbHHannover, Germany; ^3^Department of Neurology, German Centre for Vertigo and Dizziness, University Hospital MunichMunich, Germany

**Keywords:** vertigo, treatment, betahistine, “observational study,” “registry”

## Abstract

**Introduction:** Despite being a common disease, data on vertigo management in a real-world setting are scarce.

**Aims:** To provide information on the vertigo and its management in a real-world setting.

**Methods:** Data were collected from 4,294 patients with vertigo in 13 countries over 28 months via a multi-national, non-interventional observational study (the so-called REVERT registry). Data included medical history and details of anti-vertigo therapy. “Clinical global impression” (CGI) of severity (CGI-S) was assessed at baseline (V1) and then at 6 months follow-up (V2) along with CGI change (CGI-C). All variables were analyzed descriptively.

**Results:** The majority of patients were female, >40 years of age, and almost half had co-morbid cardio-vascular disease. Diagnoses were split into four categories: 37.2% “other vertigo of peripheral vestibular origin,” 26.9% benign paroxysmal positional vertigo (BPPV), 20.5% “peripheral vestibular vertigo of unknown origin,” and 15.4% Ménière’s disease (MD). Betahistine was the most commonly prescribed therapy prior to and after enrollment, and was followed by piracetam, ginkgo biloba, and diuretics. MD had the highest proportion of betahistine treated patients. Almost half of patients were “moderately ill” at V1 based on CGI-S. At V2, patient distribution moved toward “less severe illness” (91.0% improved). The greatest improvements were in the more severely ill, and those with BPPV or “other vertigo of peripheral origin.”

**Conclusion:** There was a reduction in illness severity over the course of the study, some of which is likely to be due to pharmacological intervention. Further studies are needed to confirm these results.

## Introduction

Vertigo, defined as an erroneous sense of motion and unsteadiness (Swartz and Longwell, 2005; Kuo et al., 2008a; Brandt et al., 2009; Strupp et al., 2011), is a relatively common condition, yet definitions vary and management guidelines are often contradictory. A survey in Germany, for example, reported that dizziness/vertigo had a prevalence of 22.9% in the last 12 months and an incidence of 3.1% (Neuhauser, 2007). The most commonly reported presenting symptom is dizziness (Labuguen, 2006) although symptoms can also include loss of balance, nausea (Kuo et al., 2008a), vomiting (Clark et al., 1994), light-headedness, and difficulty standing or walking (Hanley et al., 2001; Labuguen, 2006).

The most common causes of vertigo are vestibular disorders including: benign paroxysmal positional vertigo (BBPV), Ménière’s disease (MD), and vestibular neuritis (Hanley et al., 2001; Kuo et al., 2008a,b). Patients are variously affected depending on the underlying cause of disease (Hanley et al., 2001). For example, in BPPV, vertigo develops suddenly, lasts ∼1 min and is typically induced by changes of the head or body position. By contrast, in MD, attacks can last up to several hours (Saeed, 1998; Strupp and Brandt, 2008).

There is no single effective medication for vertigo and in clinical practice a combination of drugs are used, including antihistamines and anti-emetics (Della Pepa et al., 2006; Kuo et al., 2008b). Acutely administered anti-vertiginous medications can be given to treat the attack; however, these have limited benefit and no effect in those patients where episodes only last a few seconds (Swartz and Longwell, 2005; Brandt et al., 2009).

While dizziness and vertigo are often reported as common and frustrating complaints in general practice (Hanley et al., 2001; Labuguen, 2006), and can account for a significant number of consultations (Hanley et al., 2001) there is a lack of agreement on the treatment and management of vertigo both from national and regional perspectives.

Little is known about the therapies most commonly prescribed for vertigo, compliance with these therapies and their overall effect on illness. A vertigo registry was specifically established to provide information about patients with vertigo in terms of the clinical characteristics of the illness and to assess the potential economic effects of vertigo. This report covers the clinical and demographic findings from the registry.

## Materials and Methods

### Study design and patient inclusion

The Registry to Evaluate the burden of disease in VERTigo (REVERT) was established to collect data for a multi-national, non-interventional observational study of patients with vertigo. Investigators, which included Ear Nose and Throat Specialists (ENTs), Neurologists, General Practitioners, and Accident and Emergency physicians, were recruited by representatives of Solvay pharmaceuticals (now Abbott). The registry enrolled patients from 13 countries (Algeria, Czech Republic, Egypt, Germany, Hungary, Lithuania, Malaysia, Morocco, Russia, Slovenia, South Africa, Tunisia, and Ukraine) and collected data over a period of 28 months. Participating countries were selected based on willingness to participate, followed national regulations, and attained ethical approval locally. Investigators recruited their next two consecutive patients who were diagnosed with a new onset of vertigo of peripheral origin (either as first diagnosis for the enrolling physician or as a new consultation) and included those with BPPV, MD, other vertigo of peripheral vestibular origin, and/or peripheral vestibular vertigo of unknown pathology. The case-report form used for the purpose of the study did not allow to further differentiate the diagnoses of patients included in the category “other vertigo of peripheral vestibular origin.” No further inclusion criteria were defined and there were no specific instructions regarding treatment implementation, diagnostics, or on-going examination schedule to allow for real-world understanding of the complexities of vertigo diagnosis, treatment, and prognosis.

Data were collected from April 2007 to August 2009; participating investigators collected information at baseline and at 6 months follow-up. Case-report forms were used to record demographic information; diagnosis, age grouping, gender, and clinical information; significant medical history, anti-vertigo therapy, adverse events, and “clinical global impression” (CGI) score. Significant medical history included documentation of concomitant illness including cardio-vascular disease, hormonal dysfunction, neurological disorder (such as Parkinson disease, epilepsy, multiple sclerosis), cranial trauma, cancer, psychological disorder, or drug abuse. Anti-vertigo therapy included documentation of vestibular rehabilitation therapy as well as prescription of betahistine, piracetam, ginkgo biloba, benzodiazepines, diuretics, homeopathics, prochlorperazine, antihistamines, and other vertigo therapy; the case-report form also included the following question: “As a therapy of vertigo symptoms, following treatment is considered for this patient: surgery (yes/no), intratympanic gentamycin treatment (yes/no).” The CGI is a 3-item observer-rated scale used in this registry to measure illness severity (CGI-S), and global improvement or change (CGI-C) (U.S. Department of Health, 1976). The CGI-S is rated on a 7-point scale, with the severity of illness scale using a range of responses. CGI-C scores also range from 1 through to 7. CGI-S was assessed at baseline based on the patient’s description of their condition for the 2 days preceding the visit and classified as, “normal not ill at all,” “borderline ill,” “mildly ill,” “moderately ill,” “markedly ill,” “severely ill,” “among the most extremely ill patients.” Evaluation of change in the patients’ condition (CGI-C) was assessed by estimated improvement from baseline to follow-up as: “not assessed,” “very much improved,” “much improved,” “minimally improved,” “no change,” “minimally worse,” “much worse,” “very much worse,” and [“worsened” (only in French documentation)]. The classes “very much improved” “much improved” and “minimally improved” were further summarized as “improved.” The classes “very much worse” “much worse” and “minimally worse” were further summarized as “worsened.” Any serious adverse drug reactions were reported. The study was conducted according to accepted standards of good clinical practice, in agreement with the latest version of the Declaration of Helsinki and in keeping with local regulations. Informed consent was obtained from all subjects and was archived by the study investigators according to local requirements.

### Statistical analysis

All summary statistics were performed using SAS software (version 9.2). All analysis variables were analyzed descriptively. Statistical significance was not determined in this study. Discrete data were summarized presenting counts and percentages, missing values were not considered for the calculation of percentages. Analysis of CGI is based on data from patients with a baseline and follow-up visit. CGI-C were analyzed in shift tables, comparing baseline with follow-up.

## Results

### Demographics

A total of 4,294 patients were enrolled in the registry from 618 different international centers; of these, a total of 4,121 composed the first data set consisting of patients suitable for inclusion and providing data on prior medical history. Data set two consisted of 4,105 patients who provided data at baseline and contributed to descriptive statistics (Table 1) and of 3,533 (82.3%) patients who attended both baseline and follow-up visits and so provided complete information and met the requirements for inclusion in the statistical analysis.

**Table 1:** **Demographics at baseline**.

Variable	*n* (%)
**COUNTRY (*n* = 4,105)**	
Algeria	274 (6.7)
Czech Republic	559 (13.6)
Egypt	168 (4.1)
Germany	99 (2.4)
Hungary	1,320 (32.2)
Lithuania	202 (4.9)
Malaysia	354 (8.6)
Morocco	118 (2.9)
Russia	253 (6.2)
Slovenia	130 (3.2)
South Africa	34 (0.8)
Tunisia	185 (4.5)
Ukraine	409 (10.0)
GENDER (*n* = 4,079)*	
Male	1,417 (34.7)
Female	2,662 (65.3)
AGE RANGES, YEARS (*n* = 4,093)**	
≤20	29 (0.7)
21–30	181 (4.4)
31–40	437 (10.7)
41–50	799 (19.5)
51–60	1,079 (26.4)
61–70	816 (19.9)
71–80	548 (13.4)
>80	204 (5.0)
DIAGNOSIS (*n* = 4,048)***	
Ménière’s disease	625 (15.4)
Benign paroxysmal positional vertigo	1,090 (26.9)
Other vertigo of peripheral vestibular origin	1,504 (37.2)
Peripheral vestibular vertigo of unknown origin	829 (20.5)

*n = 26 missing data on gender; **n = 12 missing data on age; ***n = 57 missing data on diagnosis.

The majority of patients were female (65.3%) and over 65% were between the ages of 41 and 70 years. The mean age at diagnosis was 56.1 years (SD 15.07 range 13–98 years; *n* = 4,093); only a very small number of patients were below the age of 20 years (0.7%). The most common diagnosis was “other vertigo of peripheral vestibular origin” (37.2%), followed by BPPV (26.9%), peripheral vestibular vertigo of unknown origin (20.5%), and MD (15.4%). Diagnosis at baseline visit, when split by gender, showed approximately the same distribution, with “other vertigo of peripheral vestibular origin” being the most frequent diagnosis.

### Medical history

Any significant medical history was documented at baseline, visit 1, and is documented in Table 2. A total of 429 patients had missing data on medical history. Of the 3,676 patients providing medical history, almost half (46.3%) had cardio-vascular disease. In addition, 17.2% had hormonal dysfunction, e.g., diabetes, and 15.2% had psychiatric disorders. There were also reports of neurological disorders, cranial tumors, neoplasms, and drug/alcohol abuse. Multiple entries were possible.

**Table 2:** Patients with significant medical history at baseline (*n* = 3,676)***.

Medical history	Total *n* (%)
Cardiac/vascular disease	1,702 (46.3)
Hormonal dysfunction	634 (17.2)
Neurologic disorder	338 (9.2)
Cranial trauma	204 (5.5)
Neoplasm	133 (3.6)
Psychiatric disorder	558 (15.2)
Drug/alcohol abuse	107 (2.9)

***n = 429 missing data on medical history.

### Treatments for vertigo

Treatments prior to inclusion in the registry were recorded, with any treatment prescribed at baseline, and at follow-up.

Prior to inclusion in the registry, the most commonly prescribed therapy was betahistine (26.6%). Piracetam was used by 11.5%, ginkgo biloba by 5.7%, and diuretics by 5.3%. In addition, patients were also using benzodiazepines, calcium antagonists, neuroleptics, antihistamines, and homeopathic medications (Table 3). Co-prescription of treatments was common.

**Table 3:** **Medical therapy**.

Medical therapy	% of patients on therapy before entry	% of patients started on a therapy at baseline (visit 1)	% of patients already on therapy before visit 2	Additional % of patients who started on a therapy at visit 2
Betahistine	26.6	66.6	66.1	21.5
Piracetam	11.5	11.0	14.0	3.3
Ginkgo biloba	5.7	5.2	6.4	3.1
Other vertigo therapy	4.7	5.9	5.1	1.9
Diuretics	5.3	3.1	4.6	0.9
Benzodiazepines	4.0	2.6	3.7	1.5
Antihistamines	1.7	1.3	1.7	0.9
Neuroleptics	1.9	1.1	1.5	0.6
Calcium antagonists	2.2	0.6	1.8	0.7
Homeopathics	1.1	0.8	0.5	0.5

The administration of medicines prior to entry into the registry was also stratified by diagnosis. Again, the most commonly prescribed medication was betahistine, used in 34.8% of patients with MD (218 of 627), 24.6% of patients with BPPV (268 of 1,091), 23.6% of those with peripheral vestibular vertigo of unknown origin (196 of 832), and in 22.8% of patients with other vertigo of peripheral vestibular origin (344 of 1,512). Patients affected by MD had the highest proportion of betahistine treatment when compared with the other diagnosis groups. Patients affected by “other vertigo of peripheral origin” received piracetam more frequently (13.8%) than any other patients. Patients affected by “peripheral vestibular vertigo of unknown origin” received benzodiazepines (4.8%) as well as neuroleptics (3.2%) more frequently and ginkgo biloba less frequently (4.3%) than patients in the other treatment groups.

### Treatment for vertigo after enrollment

Prescription of a treatment after enrollment to the registry was at the discretion of the participating physician and was not mandatory. The most common treatment started at both visits was betahistine with 66.6% of subjects prescribed this drug at visit 1 and 21.5% at visit 2 (Table 3). Piracetam was reported to be prescribed in less than 15.0% of patients at each visit (Table 3).

Drug treatment at baseline and at follow-up was also stratified by diagnosis; overall, betahistine was prescribed in the majority of patients in each diagnosis group. Marginally more patients with MD received betahistine than any other diagnosis group (Table 4). By contrast, piracetam was prescribed more often in patients with “other vertigo of peripheral origin” than in any other diagnostic group.

**Table 4:** **Most frequent drug treatments by diagnosis**.

	Betahistine		Piracetam		Ginkgo biloba	
	Baseline	Follow-up	Baseline	Follow-up	Baseline	Follow-up
Ménière’s disease *n* (%) (*n* = 672)	595 (94.9)	453 (72.3)	106 (17.0)	69 (11.1)	72 (11.4)	48 (7.7)
BPPV *n* (%) (*n* = 1,091)	933 (85.6)	724 (66.3)	174 (15.9)	119 (10.9)	125 (11.4)	70 (6.4)
Other vertigo *n* (%) (*n* = 1,512)	1,377 (91.1)	1,112 (73.6)	435 (28.7)	263 (17.4)	170 (11.3)	139 (9.2)
Peripheral vestibular vertigo *n* (%) (*n* = 832)	763 (91.7)	627 (75.4)	170 (20.4)	126 (15.1)	69 (8.3)	58 (7.0)

In addition to these treatments, surgery as a therapy for vertigo symptoms was considered (but not necessarily undertaken) for 55 patients at baseline and intratympanic gentamycin treatment was also considered (but not necessarily undertaken) for 45 of the 4,121 patients at baseline.

### Clinical global impression

**Figure 1:**
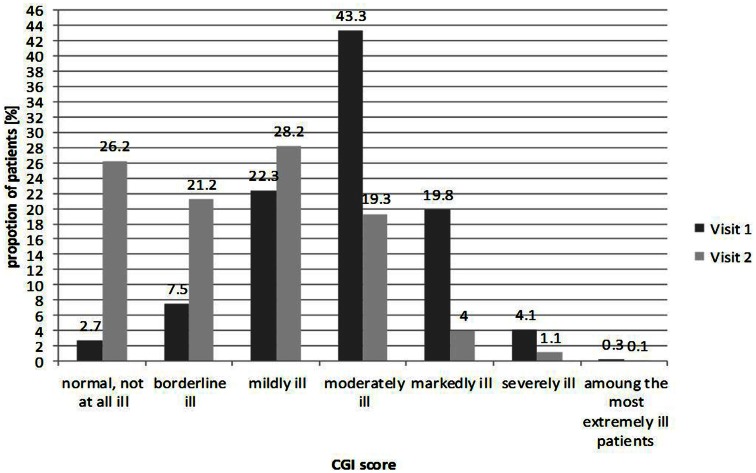
**Clinical global impression at baseline visit and follow-up (*n* = 4,192 at visit 1; *n* = 3,566 at visit 2)**.

CGI-S was documented at baseline and follow-up visit as shown in Figure 1. Almost half of patients (43.3%) were classified as “moderately ill” at baseline. Roughly one quarter of patients were classified as “mildly ill” (22.2%), one fifth as “markedly ill” (20.0%), and only a small minority of patients at either end of the distribution, evaluated as “not ill,” “severely ill,” or “extremely ill.”

By contrast, at the follow-up visit, the overall distribution moved toward less severe forms of illness, with the majority (75.0%) of patients classified as normal to mildly ill; over one quarter of patients (26.0%) were classified as “not ill,” 21.0% as “borderline ill,” and 28.0% as only “mildly ill.” Similar results were seen when CGI was assessed by gender (data not shown).

**Figure 2:**
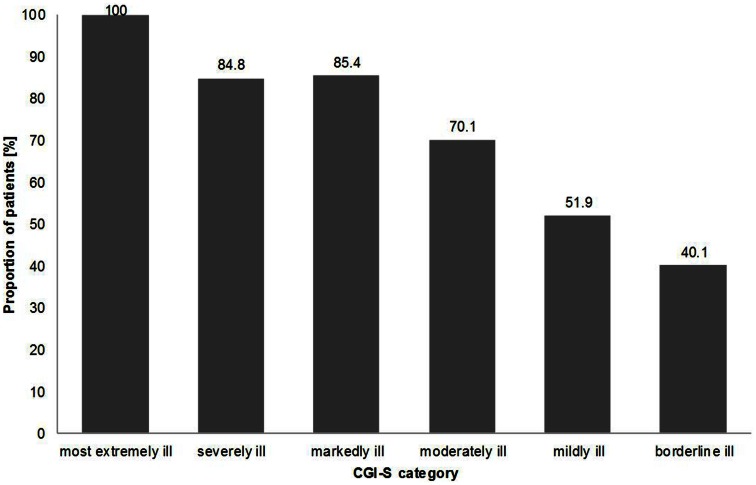
**Change in severity of illness; percentage of patients classified as “improved” at follow-up according to baseline CGI-S category**.

Data on the change of severity of the illness from baseline to follow-up showed that generally the greatest changes in status were seen in the patients originally classified as more severely or markedly ill and the greatest percentage of patients evaluated as worsening were amongst those initially classified with milder forms of illness. For example, 100.0% of patients assessed as the “most extremely ill” improved their clinical status at follow-up. However, only 40.1% of patients classified as “borderline ill,” improved at follow-up. Figure 2 illustrates the results from those patients assessed as having improved at follow-up based on the severity of their illness (CGI-S) at baseline.

Assessment of change by diagnosis suggested that the most impressive changes were seen in patients with BPPV; 66.7% of those who were “extremely ill” at baseline were “not ill at all” at 6 months follow-up, and 40.8% who were “markedly ill” at baseline were reported as “not at all ill” at follow-up. Patients with “other vertigo of peripheral origin” also showed major improvements from baseline to follow-up; however, those with MD had the least impressive changes. Table 5 documents these changes by showing the patients who were classified as having recovered completely (i.e., not at all ill) at follow-up from each diagnostic group by CGI at baseline.

**Table 5:** **Proportion of patients in each severity class having recovered during the registry according to CGI evaluation**.

CGI evaluation 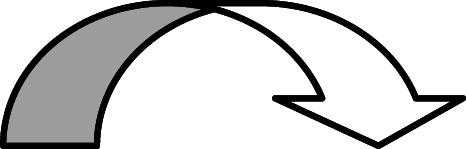		Total population (%)	Ménière’s disease (%)	BPPV (%)	Peripheral vestibular vertigo (%)	Other vertigo (%)
Change from visit 1	To visit 2	
“Among the most extremely ill patients”	“Normal (not at all ill)”	33.3	0.0	66.7	0.0	40.0
“Severely ill”		20.5	8.3	29.4	13.3	25.0
“Markedly ill”		25.9	19.9	40.8	12.3	27.7
“Moderately ill”		21.7	17.8	27.3	19.6	20.9
“Mildly ill”		27.1	35.2	35.4	25.2	20.9
“Borderline ill”		40.1	33.3	45.2	39.5	38.7

*BPPV; benign paroxysmal positional vertigo*.

**Figure 3:**
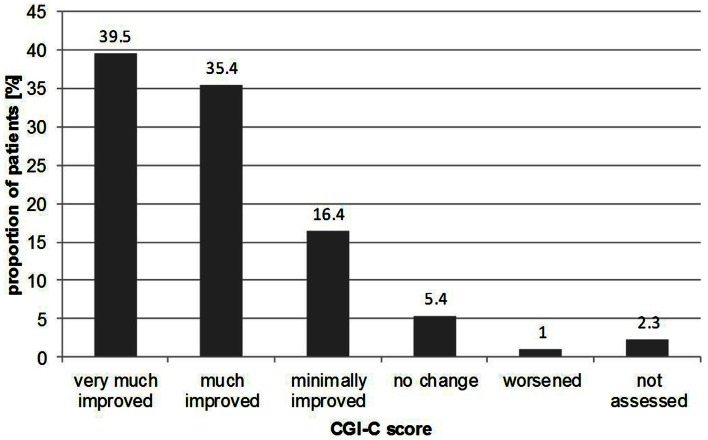
**Proportion of patients with changes in illness according to CGI-C results at follow-up**.

For CGI-C, according to the treating physician at follow-up, the majority of patients (91.0%) had improved during the 6 months from baseline to follow-up, with 39.5% “very much improved,” 35.4% “much improved,” and 16.4% “minimally improved.” Figure 3 illustrates the results from CGI-C, where the change of clinical impression showed no considerable differences in distribution according to gender with 92.4% of males “improving” compared with 90.8% of females. When stratified by age, again, no substantial differences were seen. For example, 92.0% of patients in the 31–40 year group “improved” compared with 92.1% in the 71–80 year group.

### Safety

Safety records showed one serious adverse event was reported during the duration of the registry; this was assessed to be unrelated to treatment.

## Discussion

The aim of the REVERT registry was to provide large-scale, multi-national real-world data on vertigo and its management. This included medical history (co-morbid conditions and treatment), details about treatment prescribed at first and second visit and clinical impressions about the course of the disease over a 6-month period. The majority of patients were female, over 40 years of age, and almost half had co-morbid cardio-vascular disease. While epidemiologic data on the prevalence of specific dizziness and vertigo disorders are scarce (Neuhauser, 2007), these findings are in line with other published data. For example in a national survey from Taiwan, mean age was 55 years with a ratio of 1:1.96 of men to women (Lai et al., 2011) and in a US survey of emergency room visits for dizziness, mean age was 51 years with 61.0% female (Newman-Toker et al., 2008). Medical history results from our study show the high prevalence of medical conditions, such as cardio-vascular disorders, hormonal dysfunctions, and psychiatric disorders. These results are consistent with other series which suggest that the most frequent diagnostic category in patients admitted in emergency departments for acute dizziness is oto-vestibular, but that general medical diagnoses (especially cardio-vascular) are highly prevalent in this acute care population (Newman-Toker et al., 2008). In the general practice setting, the three most common diagnoses of vertigo (accounting for 93.0% of all patient presentations) are BPPV, acute peripheral vestibulopathy (vestibular neuritis or labyrinthitis), and MD (Halmagyi and Cremer, 2000). The results from this series are similar; the most common diagnoses for vertigo being of peripheral vestibular origin and BPPV. It needs to be noted that the case-report form did not ask for migraine, which explains why neither migraine with or without aura nor vestibular migraine appear in our results.

Betahistine was the most frequently prescribed treatment in this study; it was already prescribed to 26.0% of patients prior to enrollment and was prescribed to nearly two thirds of patients at first visit (66.0%). Our results suggest that MD was the diagnosis in which betahistine was most commonly prescribed but they also show that it was being used in 86.0% of patients with BPPV. Other data on the treatment and management of vertigo in “real-life” clinical practice are limited, but a recent observational study, similar to the current study, reported that betahistine was prescribed in over half (∼56.0%) the patients at new diagnosis (Benecke et al., 2010).

Other therapies recorded in the REVERT registry included piracetam, ginkgo biloba, diuretics, benzodiazepines and antihistamines. With the exception of piracetam, these were used in less than 6.0% of patients at each observation. Piracetam is an established treatment with several indications including vertigo. It was prescribed in 14.0% of patients in this study and more often in the group of patients with “other vertigo” than in any other group. Ginkgo biloba extract is used clinically in the treatment of inner ear disorders such as hearing loss, vertigo, and tinnitus; however, recent evidence in support of its effectiveness is lacking.

At baseline, CGI-S scores suggested that almost half of patients were classified as “moderately ill” (43.0%) while 4.0% were considered to be “severely ill” and 7.5% “borderline ill.” Over the course of the REVERT registry, the severity of vertigo was reported to decrease from baseline visit to follow-up at 6 months and the proportions of patients in these groups altered accordingly. Only 19.0% were “moderately ill” at 6 months, and only 1.0% “severely ill.” Results from the CGI-C showed that 91.0% of patients were thought to have improved over the course of the registry, with nearly 40.0% of patients classified as “very much improved.” How much of this improvement was the result of treatment and how much was due to spontaneous recovery of symptoms, is not clear.

Spontaneous recovery is known to be common and reported rates of spontaneous complete resolution of BPPV range from 20 to 80% at 1 month of repositioning treatment (Hornibrook, 2011). The American Academy of Otolaryngology, Head and Neck Surgery Clinical Practice Guideline for BPPV recommends that 1 month should be the standard interval after treatment for retesting (Bhattacharyya et al., 2008). Patients who were “markedly ill” appeared to be the most likely to improve over the course of the registry, despite results suggesting that those who were “moderately ill” received the highest application rate of medical therapies. This further supports the idea that many of the patients might have had spontaneous resolution of their conditions.

This registry-based, observational study has several limitations. The drawbacks associated with such studies are well-known; invalid conclusions can result when, for example, there are insufficient quality and depth to the data, potential sources of bias, and significant missing data. The data collected during this study were limited to medical diagnosis, clinical global assessment, and treatment. The diagnoses were not validated, and there is no information on diagnosis definitions which could mean that these differed from country to country and institution to institution. The CGI is widely used and yet is also associated with several known limitations; it lacks validation, there is no specific interviewer guide available, and the response format used to assess change or severity of illness is often ambiguous (Kadouri et al., 2007). This study included over 4,000 patients at outset but nearly 18.0% were lost to follow-up. There may be several reasons for non-attendance at follow-up and spontaneous recovery could be a major cause. Change of treatment during the course of the registry cannot be tracked with any certainty and the records suggest that treatment may have been stopped and then re-started at follow-up, but precise details are lacking. Lastly, this was a multi-national study involving countries with different languages, cultures, and national approaches to health care. As a result there is likely to be a large amount of heterogeneity in the data, which again make robust conclusions difficult to interpret.

Despite these drawbacks registries are useful tools which allow for long-term, large-scale data collection in a real-world setting, and are used regularly for pharmacovigilance, safety monitoring, and to understand the epidemiology and burden of disease. Whilst patient registries cannot offer comparable robustness to controlled clinical trials, it is important to remember that real-world scenarios are uncontrolled in their nature, and well-managed registries can provide a wealth of information and valuable insight into the actual clinical experience. The results from this registry can help to inform primary care physicians; vestibular vertigo accounts for a considerable healthcare burden, which suggests that diagnosis and treatment of frequent vestibular conditions are important issues in primary care (Neuhauser et al., 2008). Currently, little is known about the drugs most commonly prescribed for vertigo, compliance with these therapies and their overall effect on illness; this study provides some evidence in this context. This study also highlights a key point in the management of these patients – i.e., that most improve. Thus, an important clinical implication is that physicians who diagnose and treat patients with vertigo can give a likely favorable prognosis.

In conclusion, our results showed that betahistine was used to treat the majority of patients in the registry and that 93.0% of patients had improved after 6 months follow-up, with nearly 40.0% of patients classified as “very much improved.” This indicates a considerable reduction in illness severity, some of which is likely to be the result of pharmacological support. Further studies are necessary to confirm these results and more consistent and up-to-date guidelines are needed for the management of patients with vertigo.

## Conflict of Interest Statement

Heike Benecke is an employee of Abbott Products GmbH, Germany. Sam Agus and Cornelia Thum are former employees of Abbott Products Operations AG, Switzerland. Michael Strupp is employed by the University Hospital Munich (Department of Neurology and IFB LM).

## References

[B1] Benecke H. Perez-Garrigues H. Bin Sidek D. Uloziene I. Kuessner D. Sondag E. (2010). Effects of betahistine on patient-reported outcomes in routine practice in patients with vestibular vertigo and appraisal of tolerability: experience in the OSVaLD study. Int. Tinnitus J. 16, 14–24 21609908

[B2] Bhattacharyya N. Baugh R. F. Orvidas L. Barrs D. Bronston L. J. Cass S. (2008). Clinical practice guideline: benign paroxysmal positional vertigo. Otolaryngol. Head Neck Surg. 139, S47–S81 10.1016/j.otohns.2008.01.018 18973840

[B3] Brandt T. Zwergal A. Strupp M. (2009). Medical treatment of vestibular disorders. Expert Opin. Pharmacother. 10, 1537–1548 10.1517/14656560902976879 19527184

[B4] Clark M. R. Sullivan M. D. Fischl M. Katon W. J. Russo J. E. Dobie R. A. (1994). Symptoms as a clue to otologic and psychiatric diagnosis in patients with dizziness. J. Psychosom. Res. 38, 461–470 10.1016/0022-3999(94)90107-4 7965935

[B5] Della Pepa C. Guidetti G. Eandi M. (2006). Betahistine in the treatment of vertiginous syndromes: a meta-analysis. Acta Otorhinolaryngol. Ital. 26, 208–215 18236637 PMC2640000

[B6] Halmagyi G. M. Cremer P. D. (2000). Assessment and treatment of dizziness. J. Neurol. Neurosurg. Psychiatr. 68, 129–134 10.1136/jnnp.68.2.129 10644773 PMC1736769

[B7] Hanley K. O’Dowd T. Considine N. (2001). A systematic review of vertigo in primary care. Br. J. Gen. Pract. 51, 666–671 11510399 PMC1314080

[B8] Hornibrook J. (2011). Benign paroxysmal positional vertigo (BPPV): history, pathophysiology, office treatment and future directions. Int. J. Otolaryngol. 2011, 835671 21808648 10.1155/2011/835671 PMC3144715

[B9] Kadouri A. Corruble E. Falissard B. (2007). The improved clinical global impression scale (iCGI): development and validation in depression. BMC Psychiatry 7:7 10.1186/1471-244X-7-7 17284321 PMC1802073

[B10] Kuo C. H. Pang L. Chang R. (2008a). Vertigo – part 1 – assessment in general practice. Aust. Fam. Physician 37, 341–347 18464964

[B11] Kuo C. H. Pang L. Chang R. (2008b). Vertigo – part 2 – management in general practice. Aust. Fam. Physician 37, 409–413 18523693

[B12] Labuguen R. H. (2006). Initial evaluation of vertigo. Am. Fam. Physician 73, 244–251 16445269

[B13] Lai Y. T. Wang T. C. Chuang L. J. Chen M. H. Wang P. C. (2011). Epidemiology of vertigo: a National Survey. Otolaryngol. Head Neck Surg. 145, 110–116 10.1177/0194599811416318a219 21493319

[B14] Neuhauser H. (2007). Epidemiology of vertigo. Curr. Opin. Neurol. 20, 40–46 10.1097/WCO.0b013e328013f432 17215687

[B15] Neuhauser H. Radtke A. von Brevern M. Lezius F. Feldmann M. Lempert T. (2008). Burden of dizziness and vertigo in the community. Arch. Intern. Med. 168, 2118–2124 10.1001/archinte.168.19.2118 18955641

[B16] Newman-Toker D. E. Hsieh Y. H. Camargo C. A. Pelletier A. J. Butchy G. T. Edlow J. A. (2008). Spectrum of dizziness visits to US emergency departments: cross-sectional analysis from a nationally representative sample. Mayo Clin. Proc. 83, 765–775 10.4065/83.7.765 18613993 PMC3536475

[B17] Saeed S. R. (1998). Fortnightly review. Diagnosis and treatment of Ménière’s disease. BMJ 316, 368–372 10.1136/bmj.316.7128.368 9487176 PMC2665527

[B18] Strupp M. Brandt T. (2008). Diagnosis and treatment of vertigo and dizziness. Dtsch. Arztebl. Int. 105, 173–180 19629221 10.3238/arztebl.2008.0173 PMC2696792

[B19] Strupp M. Thurtell M. J. Shaikh A. G. Brandt T. Zee D. S. Leigh R. J. (2011). Pharmacotherapy of vestibular and ocular motor disorders, including nystagmus. J. Neurol. 258, 1207–1222 10.1007/s00415-011-6245-0 21461686 PMC3132281

[B20] Swartz R. Longwell P. (2005). Treatment of vertigo. Am. Fam. Physician 71, 1115–1122 15791890

[B21] U.S. Department of Health. (1976). ECDEU Assessment Manual for Psychopharmacology – Revised (Washington, D.C.), 218-222. [DHEW Publ No ADM 76-338].

